# Diversity and extracellular enzymatic activities of yeasts isolated from King George Island, the sub-Antarctic region

**DOI:** 10.1186/1471-2180-12-251

**Published:** 2012-11-06

**Authors:** Mario Carrasco, Juan Manuel Rozas, Salvador Barahona, Jennifer Alcaíno, Víctor Cifuentes, Marcelo Baeza

**Affiliations:** 1 Laboratorio de Genética, Depto. de Ciencias Ecológicas, Facultad de Ciencias, Universidad de Chile, Las Palmeras 3425 Casilla, Santiago, 653, Chile

**Keywords:** Antarctic yeasts, Psychrophilic-psychrotolerant yeasts, Extracellular enzyme activities, rDNA yeast identification

## Abstract

**Background:**

Antarctica has been successfully colonized by microorganisms despite presenting adverse conditions for life such as low temperatures, high solar radiation, low nutrient availability and dryness. Although these “cold-loving” microorganisms are recognized as primarily responsible for nutrient and organic matter recycling/mineralization, the yeasts, in particular, remain poorly characterized and understood. The aim of this work was to study the yeast microbiota in soil and water samples collected on King George Island.

**Results:**

A high number of yeast isolates was obtained from 34 soil and 14 water samples. Molecular analyses based on rDNA sequences revealed 22 yeast species belonging to 12 genera, with *Mrakia* and *Cryptococcus* genera containing the highest species diversity*.* The species *Sporidiobolus salmonicolor* was by far the most ubiquitous, being identified in 24 isolates from 13 different samples. Most of the yeasts were psychrotolerant and ranged widely in their ability to assimilate carbon sources (consuming from 1 to 27 of the 29 carbon sources tested). All species displayed at least 1 of the 8 extracellular enzyme activities tested. Lipase, amylase and esterase activity dominated, while chitinase and xylanase were less common. Two yeasts identified as *Leuconeurospora sp.* and *Dioszegia fristingensis* displayed 6 enzyme activities.

**Conclusions:**

A high diversity of yeasts was isolated in this work including undescribed species and species not previously isolated from the Antarctic region, including *Wickerhamomyces anomalus*, which has not been isolated from cold regions in general. The diversity of extracellular enzyme activities, and hence the variety of compounds that the yeasts may degrade or transform, suggests an important nutrient recycling role of microorganisms in this region. These yeasts are of potential use in industrial applications requiring high enzyme activities at low temperatures.

## Background

Permanently cold environments are widely distributed on Earth, and include the Polar Regions, mountains and deep-sea environments. Despite presenting adverse conditions for life, such as freezing temperatures, low nutrient availability, high water viscosity and reduced membrane fluidity, these environments have been successfully colonized by the three domains of life [1]. Cold-adapted microorganisms can grow at 0°C and are classified as psychrophilic if their optimum and maximum temperatures for growth are ≤15°C and ≤ 20, respectively, or as psychrotolerant (psychrotrophic) if their maximum temperature for growth is above 20°C [2,3]. Such microorganisms have adapted their vital cellular processes to thrive in cold environments [4]. They make essential contributions to nutrient recycling and organic matter mineralization, via a special class of extracellular enzymes known as “cold-adapted” or “cold-active” enzymes [5]. Because these enzymes have a higher catalytic efficiency than their mesophilic counterparts at temperatures below 20°C and display unusual substrate specificities, they are attractive candidates for industrial processes requiring high enzymatic activity at low temperatures. Cold-adapted enzymes include amylase, cellulase, invertase, inulinase, protease, lipase and isomerase, which are used in the food, biofuel and detergent industries [6]. Largely because of their potential in biotechnological applications, cold-adapted microorganisms have become increasingly studied in recent years, yet remain poorly understood. Of the microorganisms most isolated and studied from cold environments, the majority are bacteria, while yeasts constitute a minor proportion [1].

Antarctica is considered the coldest and driest terrestrial habitat on Earth. It is covered almost totally with ice and snow, and receives high levels of solar radiation [7]. The Sub-Antarctic region, including the Shetland South Archipelago, has warmer temperatures, the soils close to the sea are free of snow/ice and receive significant quantities of organic material from marine animals; however, they are subject to continuous and rapid free-thaw cycles, which are stressful and restrictive to life [8]. Although the first report of Antarctic yeasts was published 50 years ago [9] current reports have focused on cold-tolerant Bacteria and Archaea, with yeasts receiving less attention. Yeasts dwelling in Antarctic and Sub-Antarctic maritime and terrestrial habitats belong mainly to the *Cryptococcus*, *Mrakia, Candida* and *Rhodotorula* genera [10-12]. In a recent work, 43 % of Antarctic yeast isolates were assigned to undescribed species [13], reflecting the lack of knowledge regarding cultivable yeasts that colonize the Antarctic soils. Yet these organisms constitute a valuable resource for ecological and applied studies.

This work describes the isolation of yeasts from terrestrial habitats of King George Island, the major island of the Shetland South archipelago. The yeast isolates were characterized physiologically and identified at the molecular level using the D1/D2 and ITS1-5.8S-ITS2 regions of rDNA. In addition, the ability of the yeasts to degrade simple or complex carbon sources was evaluated by analyzing their extracellular hydrolytic enzyme activities. Characterizing these enzyme activities may enhance the potential of the yeasts in industrial applications.

## Results

### Isolation of psychrophilic and psychrotolerant yeasts

The 34 soil and 14 water samples obtained from different areas of King George Island were processed as described in the Methods section. The suspensions obtained from each soil samples were seeded onto nutritive plates, and incubated in triplicate over a range of temperatures (4, 10, 15 and 22°C). After 30–90 days of incubation, approximately 30 to 60 yeast-like colonies developed on each plate. In contrast, no colonies or low colony numbers (4 to 8) appeared on plates from water samples. Because large numbers of isolates were obtained, isolates were grouped according to their isolation growth temperature and colony characteristics such as pigmentation, texture, elevation and size. Among the 64 groups, several differed only by isolation growth temperature. These isolates were grown at different temperatures and re-grouped according to macromorphological characteristics at their optimal growth temperature. In this way, 35 groups were ultimately generated. Several isolates from each group (at least one isolate per sampling site; a total of 78 isolates) were selected for molecular and biochemical analyses.

### Molecular identification of yeasts

The chromosomal DNA was purified from cultures of each yeast isolate and the D1/D2 region of 26S rDNA and the ITS1-5.8S- ITS2 (hereafter designated the ITS region for simplicity) regions of the rDNA were amplified by PCR. The amplicons obtained were purified from gels and sequenced on both strands. Isolates showing 100% identity in both rDNA sequences were grouped and their DNA sequences were submitted to GenBank under the accession numbers listed in Table 1. Species identification was performed by comparison with the GenBank references, using as criterion the Blast-hits with ≤ 0.5% difference with the query [14]. In 84% of the isolates the closest Blast-hits obtained for both rDNA sequences were coincident. When this was not the case, the D1/D2 results were used for identification because they yielded higher identity percentages than did the ITS (see Additional file 1). 76% of the isolates could be identified to species level by this molecular analysis. 22 species belonging to12 genera were identified, of which 80 and 20% were Basidiomycetes and Ascomycetes, respectively. The genera containing the highest number of species were *Mrakia* (5 species) and *Cryptococcus* (4 species). However, the species *Sporidiobolus salmonicolor* was the most abundant, being identified in 24 isolates from 13 different sampling sites. *Mrakia gelida* was the only yeast species present in both water and soil samples. Of the three isolates identified as *Leuconeurospora sp*., two of them (T11Cd2 and T27Cd2) possessed identical D1/D2 and ITS sequences, both of which differed from the third (T17Cd1) by 0.7%. However, the macromorphological characteristics of the three isolates, including pigmentation, differed markedly under identical culture conditions (see Additional file 2). Because of these discrepancies, the molecular and morphological analyses were repeated several times, but the results were highly consistent. The carbon source assimilation pattern also differed between the isolates, as will be discussed later.

**Table 1:** Molecular identification of yeast isolates

**Sample**	**ITS1-5.8S-ITS2**		**D1/D2**		**Identification**
	**Accession**	**Closest match**	**Accession**	**Closest match**	
sea water	JQ857022	*Candia sake* (AJ549822)	JQ856998	*Candida sake* (AJ507662)	*Candida sake*
soil	JQ857023	*Cryptococcus terricola* (FN298664)	JQ856999	*Cryptococcus terricola* (AM039670)	*Cryptococcus sp.*
soil	JQ857024	*Cryptococcus gastricus* (AF145323)	JQ857000	*Cryptococcus gastricus* (AF137600)	*Cryptococcus gastricus*
soil	JQ857026	*Metschnikowia australis* (JN197598)	JQ857002	*Metschnikowia australis* (U76526)	*Metschnikowia sp*
soil	JQ857027	*Mrakia robertii* (AY038829)	JQ857003	*Mrakia robertii* (EF643726)	*Mrakia robertii*
soil	JQ857028	*Mrakia blollopis* (AY038828)	JQ857004	*Mrakia blollopis* (AY038828)	*Mrakia blollopis*
soil	JQ857031	*Cryptococcus watticus (*FJ473373*)*	JQ857007	*Holtermanniella watticus* (FJ748666)	*Holtermanniella watticus*
soil	JQ857033	*Dioszegia crocea* (AF444406)	JQ857009	*Dioszegia crocea* (HQ256888)	*Dioszegia sp*
soil	JQ857034	*Leucosporidium drummii* (FN908919)	JQ857010	*Leucosporidiella fragaria* (DQ513270)	*Leucosporidiella fragaria*
soil	JQ857038	*Dioszegia fristingensis* (EU070927)	JQ857014	*Dioszegia fristingensis* (JN400789)	*Dioszegia fristingensis*
	JQ857039	*Dioszegia fristingensis* (EU070927)	JQ857014	*Dioszegia fristingensis* (JN400789)	*Dioszegia fristingensis*
soil	JQ857025	*Cryptococcus victoriae* (HQ717406)	JQ857001	*Cryptococcus victoriae* (JN544032)	*Cryptococcus victoriae*
soil	JQ857032	*Rhodotorula glacialis* (EF151250)	JQ857008	*Rhodotorula glacialis* (EF643741)	*Rhodotorula glacialis*
soil	JQ857035	*Mrakia gelida* (AF144485)	JQ857011	*Mrakia robertii* (EF643731)	*Mrakia sp.*
				*Mrakia frigida* (DQ513285)	
melt water, soil	JQ857036	*Mrakia gelida* (GQ911545)	JQ857012	*Mrakia gelida* (GQ911518)	*Mrakia gelida*
soil	JQ857037	*Rhodotorula glacialis* (EF151250)	JQ857013	*Rhodotorula glacialis* (AB671326)	*Rhodotorula glacialis*
soil	JQ857040	*Pseudeurotium bakeri* (GU934582)	JQ857015	*Leuconeurospora pulcherrima* (FJ176884)	*Leuconeurospora sp.*
soil	JQ857041	*Pseudeurotium bakeri* (GU934582)	JQ857016	*Leuconeurospora pulcherrima* (FJ176884)	*Leuconeurospora sp.*
melt water	JQ857021	*Wickerhamomyces anomalus* (JF416789)	JQ856997	*Wickerhamomyces anomalus* (JN180956)	*Wickerhamomyces anomalus*
soil	JQ857030	*Cryptococcus gilvescens* (AF444380)	JQ857006	*Cryptococcus gilvescens* (EF643719)	*Cryptococcus gilvescens*
soil	JQ857018	*Mrakia psychrophila* (EU224267)	JQ856994	*Mrakia psychrophila* (EU224266)	*Mrakia psychrophila*
soil	JQ857029	*Rhodotorula laryngis* (AB078500)	JQ857005	*Rhodotorula laryngis* (DQ640477)	*Rhodotorula laryngis*
soil	JQ857017	*Glaciozyma antarctica* (AY033637)	JQ856993	*Glaciozyma antarctica* (AY040642)	*Glaciozyma antarctica*
soil	JQ857019	*Leucosporidiella creatinivora* (AF444629)	JQ856995	*Leucosporidiella creatinivora* (AF189925)	*Leucosporidiella creatinivora*
soil	JQ857020	*Sporidiobolus salmonicolor* (AF444611)	JQ856996	*Sporidiobolus salmonicolor* (EU596439)	*Sporidiobolus salmonicolor*

### Physiological characteristics and extracellular enzyme activities

The isolates were grown at six temperatures (range 4 to 37°C). Almost 70% of the yeast isolates could grow at 22°C or higher, and generally grew optimally at 15°C (38%) or 22°C (31%) (Table 2). These results were accounted for in the physiological characterizations of the strains. The isolates identified as *Candida sake*, *Wickerhamomyces anomalus* and the four *Mrakia* species, tested positive in glucose fermentation assays. The yeast isolates were tested for the assimilation of 29 different carbon sources (for the detailed results see Additional file 3). Besides glucose, the yeasts primarily consumed D-xylose, D-melezitose, D-saccharose, D-trehalose and 2-ketogluconate, while lactose, levulinic acid and erythritol were less assimilated. Some yeasts could assimilate glucose alone (*Glaciozyma antarctica,* formerly *Leucosporidium antarcticum*), but others assimilated as many as 27 carbon sources (*Cryptococcus victoriae* and *Mrakia sp.*). The assimilation tests were performed for the isolates obtained from different sampling sites and identified molecularly as the same yeast species, with concordant results in most cases. However, the two isolates identified as *Mrakia psychrophila* differed in their assimilation of rhamnose and in the esculin test, while three isolates identified as *Leuconeurospora sp.,* two of which were identical at molecular level, differed significantly in their utilization of seven carbon sources. For those isolates that were molecularly identified to genera level only, the carbon assimilation profiles supported their differentiation from the closest Blast-hits in each case: *Cryptococcus sp.* differed from *Cr. terricola* (98.2% identity) in the assimilation of L-arabinose, trehalose, lactose, L-rhamnnose, L-sorbose and glucosamine; *Mrakia sp*. differed from *M. frigida* (99.7% identity) in the assimilation of maltose, ribose, erythritol and glucosamine, and from *M. robertii* (99.7% identity) in the assimilation of glycerol and erythritol; *Dioszegia sp.* differed from *D. crocea* (99.3% identity) in assimilation of raffinose, mellibiose and glycerol.

**Table 2:** Growth temperatures and extracellular enzyme activities of yeast isolates

**Yeast species**	**Temp.**	Enzyme activities halo (mm^*^)							
	**°C**	**Ami**	**Cel**	**Est**	**Lip**	**Pro**	**Pec**	**Chi**	**Xyl**
*C. sake*	4-22 (22)	-	-	-	1	-	-	-	-
*Cr. gastricus*	4-22 (22)	2	1	2	1	-	-	-	-
*Cr. gilvescens*	4-22 (22)	2	-	-	1	1	-	-	-
*Cr. victoriae*	4-15 (15)	-	4	5	2	-	-	-	-
*Cryptococcus* sp*.*	4-22 (15)	2	-	-	1	1	-	-	-
*D. fristingensis* (T11Df)	4-22 (22)	7	4	-	1	-	7	2	3
*D. fristingensis* (T9Df1)	4-22 (22)	3	-	6	1	-	-	-	-
*Dioszegia sp.*	4-15 (15)	7	-	6	-	-	6	-	-
*G. antarctica*	4-15 (10)	-	-	2	-	-	-	-	-
*H. watticus*	4-37 (30)	2	2	-	-	-	-	-	-
*Le. creatinivora*	4-22 (22)	-	-	3	1	-	-	-	-
*Le. fragaria*	4-22 (22)	-	2	2	1	-	3	-	-
*Leuconeurospora sp.* (T11Cd2)	4-22 (15)	2	-	6	-	-	-	-	-
*Leuconeurospora sp.* (T17Cd1)	4-22 (15)	-	4	3	2	1	6	2	-
*Leuconeurospora sp.* (T27Cd2)	4-22 (15)	-	2	2	1	1	-	2	-
*M. blollopis*	4-22 (15)	1	8	3	-	-	-	-	-
*M. gelida*	4-15 (10)	2	2	-	1	2	-	-	-
*M. psychrophila*	4-15 (10)	-	10	7	-	-	3	1	-
*M. robertii*	4-15 (15)	2	2	-	1	-	3	-	-
*Metschnikowia* sp.	4-22 (10)	-	-	-	1	-	2	1	-
*Mrakia sp.*	4-15 (15)	2	2	-	1	-	-	-	-
*Rh. glacialis*	4-15 (15)	2	-	2	1	-	1	-	-
*Rh. glacialis*	4-22 (10)	2	-	-	1	-	2	-	-
*Rh. laryngis*	4-30 (30)	-	-	4	2	-	2	-	-
*Sp. salmonicolor*	4-30 (22)	-	-	-	2	1	6	2	-
*W. anomalus*	4-37 (30)	-	1	2	2	5	3	-	-

The temperature of optimal growth is given in parenthesis. Ami, amilase; Cel, cellulase; Est, esterase; Lip, lipase; Pro, protease; Pec, pectinase; Chi, chitinase; Xyl, xylanase. *Measured from the edge of the colony to limit of the halo.

To estimate the ability of the yeasts to utilize nutrients in their natural environment, they were initially characterized for the production of 8 extracellular enzyme activities. As shown in Table 2, all yeasts displayed at least one enzyme activity, which further enhances their potential for biotechnological/industrial exploitation. The majority exhibited 2 to 4 enzyme activities, while two exceptional isolates exhibited 6 enzyme activities: *Leuconeurospora sp.* (T17Cd1) (cellulase, esterase, lipase, protease, pectinase and chitinase) and *Dioszegia fristingensis* (T11Df) (amylase, cellulase, lipase, pectinase, chitinase, and xylanase). The most common enzyme activities in the yeast isolates were esterase and lipase, while the least common was xylanase, demonstrated only by *D. fristingensis*. The three isolates molecularly identified as *Leuconeurospora sp.* (T17Cd1, T11Cd2 and T27Cd2) showed important differences in their enzyme activities, as was also observed in the isolates identified as *D. fristingensis* (T9Df1 and T11Df).

## Discussion

Approximately 70% of the isolated yeasts could grow at temperatures above 20°C, and 16% of them were able to grow at ≥30°C. The predominance of psychrotolerant fungi in cold environments has been previously noted, and is attributable to seasonal and local increases in soil temperature due to solar radiation [2]. In our study, the temperature measured in situ at the different sampling sites ranged from 0 to 11.9°C, but temperatures up to 20°C have been reported in this region [15-17]. The main obstacle to assessing the yeast communities in Antarctic regions is the scant knowledge regarding their environmental and nutritional requirements. Because the yeast populations/species inhabiting terrestrial and aquatic environments can colonize specific niches, no appropriate method exists for efficiently isolating all species [18]. In this work the yeasts were isolated using rich media supplemented with glucose, because almost all known yeasts can assimilate this sugar [19]. However, this culture condition could favor the proliferation of yeasts with high metabolic rates, to the detriment of slow-growing yeasts. Nevertheless, large numbers and high species diversity were attained in this study (22 species from 12 genera). Cold-loving yeasts have been isolated mainly from the Antarctic and the Arctic, and from European and South American glaciers [10]. In all of these environments, the most ubiquitous species are *Rhodotorula laryngis* and *Cr. victoriae.* On the other hand, *C. sake, D. fristingensis, G. antarctica* and *Sp. salmonicolor* have been isolated only in the Southern Cone (South American glaciers and Antarctica). This work reports for the first time the isolation of *Cryptococcus gastricus, Cryptococcus gilvescens, D. fristingensis* and *Leucosporidiella creatinivora* from an Antarctic region. Also isolated was *W. anomalus*, which is not generally found in cold regions.

During molecular analysis of the yeasts, most isolates assigned to the same species possessed identical D1/D2 and ITS sequences. Thus, combining these rDNA regions is a useful technique for rapid identification and typing of yeasts, as others have suggested [14,20,21]. However, the isolates identified as *Leuconeurospora sp.* were 0.7% and 0.9% different in their D1/D2 (578 bp) and ITS (534 bp) sequences, respectively. Similarly, the isolates identified as *D. fristingensis* exhibited identical D1/D2 (456 bp) sequences, but their ITS (479 bp) sequences were markedly different (4.4%), and their overlap was punctuated with several gaps. Furthermore, given the physiological differences between isolates that are identical or similar at molecular level, strongly support that the definitions of yeast species must be supplemented by classical characterizations.

Most yeast isolates showed lipase activity, consistent with a previous report in which all of the filamentous fungi from Antarctica displayed this activity [22]. Among the “cold loving” yeasts, lipase activity has been described in *Pseudozyma antarctica*[23], *Leucosporidium antarcticum*[24] and in species of *Cryptococcus* and *Rhodotorula*[25]. Unlike this last-mentioned study, we detected lipase activity in *R. laryngis* also. Lipase activity has also been described in *W. anomalus* from tropical environments [26]. The least common extracellular activity was xylanase, observed only in the *D. fristingensis* isolate. Although this activity has been previously described in *Cryptococcus* species [27,28], no xylanase activity was observed in the *Cryptococcus* isolates identified here. Consistent with our results, protease, amylase and esterase extracellular activities have been reported in several yeast species isolated from cold and tropical environments [24-26,29-33]. However, we present the first report of extracellular amylase activity in *Le. creatinivora*, *H. watticus*, *Leuconeurospora sp.* and *D. fristingensis*. In addition to *Mrakia* and *Rhodotorula* species, for which extracellular pectinase activity has been described [33], we detected pectinase activity in species of *Wickerhamomyces, Metschnikowia, Dioszegia, Leucosporidiella* and *Candida.* All *Mrakia* species isolated in this work showed cellulase activity, which has been previously described in *Mrakia frigida* isolated from King George Island [34]; furthermore, this activity was observed in *Cryptococcus* and *Dioszegia* species, contrary to a previous report [25]. Extracellular chitinase activity has been reported in *Cryptococcus* species [26], but here we observed this activity in *M. psychrophila*, *Sp. salmonicolor*, *Metschnikowia sp.*, *Leuconeurospora sp.* and *D. fristingensis*. We detected cellulase and chitinase activities in yeasts species that have not been described from cold regions, probably because our sampling sites included areas with vegetation and animal contact and/or were located close to the sea. Cellulose is one of the most abundant carbohydrates produced by plants [35] and chitin is the most abundant renewable polymer in the ocean, where it constitutes an important source of carbon and nitrogen [36]. Furthermore, significant quantities of lipids exist in phytoplankton [37] and in sediments of this region [38], which can explain the high incidence of lipase activity found in the yeasts. All of the extracellular enzyme activities analyzed in this work are potentially useful to industry: amylases in food processing, fermentation and pharmaceutical industries; cellulases and pectinases in textiles, biofuel processing and clarification of fruit juice; esterase in the agro-food industries; lipases and proteases in food and beverage processing, detergent formulation and environmental bioremediations; chitinases in biocontrol and treatment of chitinous waste; xylanase as a hydrolysis agent in biofuel and solvent industries [10,39-41].

## Conclusions

Similar to previous reports of microorganisms isolated from cold environments, the yeasts isolated in this work are predominately psychrotolerant. Rapid identification/typing of yeasts was achieved through the use of D1/D2 and ITS regions; however, other physiological and biochemical tests are required for accurate species/strains definition. The diversity of extracellular enzyme activities in the yeasts, and hence the diversity of compounds that may be degraded/transformed, reflects the importance of the yeast community in nutrient recycling in the Antarctic regions. In addition, studies about the adaptation of the different yeast species to adverse conditions (temperature, freeze-thaw, UV radiation, nutrient availability, competence, etc.) could shade light on the evolution of molecular mechanisms (carbon metabolisms, cell wall and protein structure, etc.), which are implicated in facilitating that accommodation. As an example, changes in protein structure are fundamental to allow conformation of the cytoskeleton, enzyme activity, etc. The Antarctic yeast isolates may potentially benefit industrial processes that require a high enzymatic activity at low temperatures, including bread, baking, textile, food, biofuel and brewing industries.

## Methods

### Sampling sites

**Figure 1:**
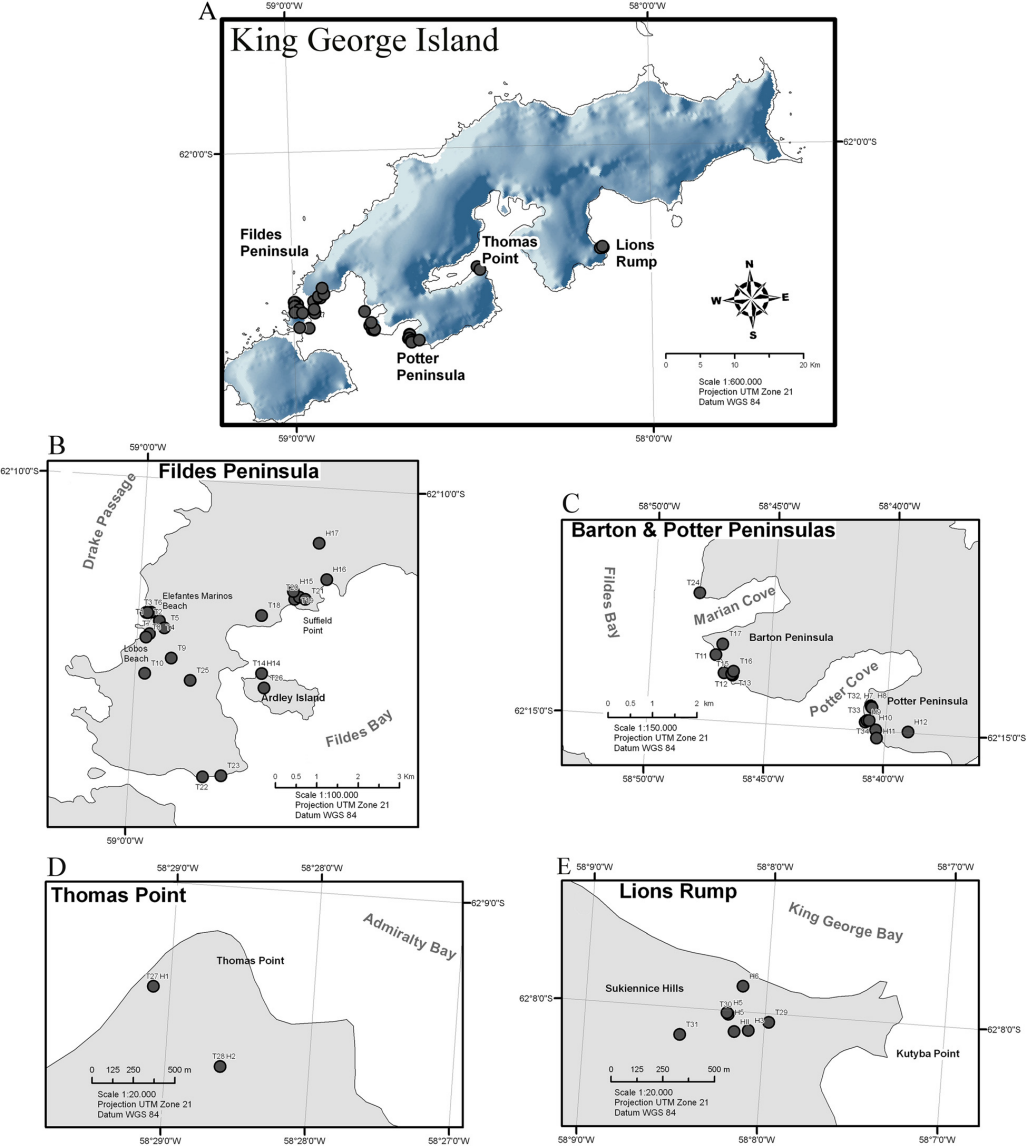
**A. Sample site locations on King George Island.B** - **E,** Zoomed-in details of the principal sampling zones. Collection sites of soil and water samples are marked with T# and H#, respectively.

All sampling sites were located on King George Island (62°02^′^S 58°21^′^W/62.033°S 58.35°W), the major island of the Shetland South Archipelago (Figure 1). A total of 34 soil and 14 water samples were collected in January of 2009. The temperature and altitude of the sampling sites varied from 0 to 11.9°C and from 0 to 182 m, respectively. For soil samples, sterile 50 ml tubes were filled with soil, sealed and stored at −20°C. For water samples, 200–500 ml of water were collected from terrestrial sources and processed in situ using the 55-PLUS™ MONITOR system (Millipore, Billerica, MA, USA,) with cellulose filter for yeasts and molds, as specified by the manufacturer. The dishes were then stored at 4°C until processing.

### Sample processing, yeast cultivation and isolation

Five grams of each soil sample was suspended in 5 ml of sterile water by vigorous agitation on a vortex for 10 min. Following decantation of the coarse particulate material, 200 μl of the suspension was seeded onto plates containing YM medium (0.3% yeast extract, 0.3% malt extract, 0.5% peptone) supplemented with 2% glucose and 100 μg/ml chloramphenicol (YM-cm). The plates were incubated at 4, 10, 15 and 22°C. Duplicate of water sampling dishes were incubated at 4 and 10°C. The plates were incubated for 3 months and periodically inspected for colony development. Once a colony became visible, it was immediately transferred to fresh YM-cm plates and incubated at the same temperature as the source-plate. The procedure was repeated for each soil sample to maximize the number of isolates.

Long-term preservation of the yeast isolates was achieved via two methods; the gelatin drop method [42,43] and cryopreservation at −80°C in 30% glycerol.

### Determination of growth temperatures and carbon source assimilation

Yeast growth at different temperatures was assessed by a method based on comparison of colony sizes on solid media, which is applicable to the determination of minimum inhibitory concentration in yeasts [44]. The yeasts were seeded onto YM plates, incubated at 4, 10, 15, 22, 30 and 37°C, and the colony sizes were recorded daily. For each yeast at each temperature, a plot of colony size vs. incubation time was constructed; the temperatures at which colony diameter increased significantly were considered as positive for growth, while the temperature at which the slope changed most rapidly was considered as the “best” or “optimal” for the growth. Glucose fermentation test were performed using a Durham tube. The assimilation of 29 different carbon sources was determined using the API ID 32C gallery (bioMérieux, Lyon, France) as specified by the manufacturer. Briefly, a colony portion was suspended in 400 μl of sterile water. Following adjustment to A_600nm_≈0.5 (equivalent to 2 McFarland standard), 250 μl of the suspension was added to an ampule of api C medium. Each well of the strip was seeded with 135 μl of this final suspension and incubated in a humid chamber. The turbidity (+) or lack thereof (−) in the wells was used as indicator of growth and was determined by visual inspection relative to the negative control well. A bionumber code was obtained from the data using the apiweb™ software.

### DNA extraction, amplification, sequencing and analysis

50 ml of each yeast culture (A_600nm_ = 0.6 to 0.8) was centrifuged at 7,000 x g for 10 min, the pellet was suspended in 5 ml of TE buffer and 300 μl aliquots of the cellular suspension were mixed with 250 μl of 0.5 mm diameter glass beads, vortexed for 10 min and centrifuged at 12,000 x g for 5 min. The DNA was obtained from 300 μl of the supernatant using the Wizard Genomic DNA Purification kit (Promega, Madison, USA) as specified by the manufacturer. The concentration and integrity of the DNA samples were analyzed by electrophoresis in 1.5% agarose gels. The D1/D2 and ITS1-5.8S-ITS2 regions of rDNA were amplified with the primers pairs F63/LR3 [45] and ITS1/ITS4 [46], respectively, using *Taq* polymerase (Fermentas International INC.) in thermal cyclers (Applied Biosystems). The resulting amplicons were separated by electrophoresis in 1.5% agarose gels immersed in TAE buffer containing ethidium bromide (0.5 μg/ml) and were purified from the gels as described in Boyle and Lew [47]. Most of the nucleotide sequences were determined using the sequencing service of Macrogen INC. In some cases, the DNA Sequencing Kit Dynamic Termination Cycle (Amersham Biosciences Limited) and a Genetic analyzer 3100 Avant automatic sequencer (Applied Biosystem) were used. The sequences were analyzed using the Geneious Pro 5.4.5 software (Biomatters, Auckland, New Zealand).

### Extracellular enzyme activity assays

All assays were performed on solid YM medium supplemented with 2% glucose (unless otherwise specified) and the appropriate substrate for enzyme activity. The plates were incubated at the optimal growth temperature of the individual yeast isolate, and the enzyme activities determined as described below.

*Amylolytic activity*. The cells were grown in medium containing 0.2% soluble starch. The plates were flooded with 1 ml of iodine solution, and positive activity was defined as a clear halo around the colony on a purple background [48].

*Cellulase activity*. The cells were grown in medium supplemented with 0.5% carboxymethylcellulose [49]. The plates were flooded with 1 mg/ml of Congo red solution, which was poured off after 15 min. The plates were then flooded with 1 M NaCl for 15 min. Positive cellulase activity was defined as a clear halo around the colony on a red background [50].

*Chitinase activity.* The cells were grown in medium containing 2.5% purified chitin. Chitinase activity was indicated directly by the presence of a clear halo around the colony [48].

*Lipase activity*. The cells were grown in medium containing 1% tributyrin. Lipase activity was indicated by a clear halo around the colony [51].

*Protease activity*. The cells were grown in medium supplemented with 2% casein at pH 6.5. Protease activity was indicated by the presence of a white precipitate around the colony [49].

*Xylanase activity.* The cells were grown in medium supplemented with 0.5% xylan [52]. Xylanase activity was indicated by a clear halo around the colony.

*Pectinase activity.* The cells were grown in 0.67% YNB medium, pH 7.0, containing 1% pectin [26]. The plates were flooded with 1% hexadecyltrimethylammonium bromide, and activity was indicated by a clear halo around the colony on a red background [48].

*Esterase activity.* The cells were grown in medium composed of 1% bacto peptone, 0.5% NaCl, 0.4% CaCl_2_*2H_2_O and 1% Tween 80 [53], and esterase activity was indicated by a white precipitate around the colony.

## Competing interests

The authors declare that they have no competing interests.

## Authors' contributions

MC, isolation and molecular characterization of yeasts; JMR, isolation and biochemical characterization of yeasts; SB, collection of water and soils samples; VC and JA, participated in the study design; MB conceived the study and participated in its design and coordination; and MB, JA and VC wrote the manuscript. All authors approved the final manuscript.

## Supplementary Material

Additional file 1 **Molecular identification of yeast isolates obtained in this work. Summary of Blast search results obtained for D1/D2 and ITS1-5.8S-ITS2 rDNA sequences.** The closets Blast-hits corresponding to uncultured yeasts were not considered. Click here for file

Additional file 2 **Colony morphology of *Leuconeurospora sp *. isolates.** Yeasts were cultivated on YM plates supplemented with glucose. The isolates T11Cd2 and T27Cd2 possess identical D1/D2 and ITS sequences, yet are morphologically different. Click here for file

Additional file 3 **Carbon source assimilation by yeast isolates obtained in this work.** Determinations were performed using the API ID 32C gallery (bioMérieux, Lyon, France) according to manufacturer′s instructions. Gal, D-galactose; Sac, D-sucrose; Nag, N-acetyl-glucosamine; Lat, lactic acid; Ara, L-arabinose; Cel, D-cellobiose; Raf, D-raffinose; Mal, maltose; Tre, D-trehalose; 2kg, 2-ketoglutamate; Mdg, Methyl-αD-glucopiranoside; Man, D-mannitol; Lac, D-lactose; Ino, Inositol; Sor, D-sorbitol; Xyl, D-xylose; Rib, D- ribose; Gly, Gycerol; Rha, L-rhamnnose; Ple, pallatinose; Ery, erytritol; Mel, mellibiose; Grt, glucoronate; Mlz, D-mellicitose; Gnt, gluconate; Lvt, levulinic acid; Glu, D-glucose; Sbe, L-sorbose; Gln, glucosamine. +, assimilation; -, no assimilation. Determinations for each yeast were performed twice. Click here for file
