# Supplementary material for: Diversity and extracellular enzymatic activities of yeasts isolated from King George Island, the sub-Antarctic region
Source: BMC Microbiol. 2012 Nov 6;12:251. doi: 10.1186/1471-2180-12-251 (PMC3499239; doi:10.1186/1471-2180-12-251)
Supplement: Additional file 1 — Molecular identification of yeast isolates obtained in this work. Summary of Blast search results obtained for D1/D2 and ITS1-5.8S-ITS2 rDNA sequences. The closets Blast-hits corresponding to uncultured yeasts were not considered. [file 1471-2180-12-251-S1.pdf]

Nº of isolates	Sites of isolation	ITS1-5.8S-ITS2			D1/D2			Identification
		Accession	Closest match	Overlap (%)	Accession	Closest match	Overlap (%)	
1	H14	JQ857022	*Candia sake* (AJ549822)	410/411 (99.8)	JQ856998	*Candida sake* (AJ507662)	297/298 (99.7)	*Candida sake*
1	T15	JQ857023	*Cryptococcus terricola* (FN298664)	612/618 (99.0)	JQ856999	*Cryptococcus terricola* (AM039670)	603/614 (98.2)	*Cryptococcus sp*
1	T23	JQ857024	*Cryptococcus gastricus* (AF145323)	611/611 (100)	JQ857000	*Cryptococcus gastricus* (AF137600)	529/530 (99.8)	*Cryptococcus gastricus*
1	T1	JQ857026	*Metschnikowia australis* (JN197598)	352/355 (99.2)	JQ857002	*Metschnikowia australis* (U76526)	529/536 (98.7)	*Metschnikowia sp*
1	T15	JQ857027	*Mrakiarobertii* (AY038829)	613/614 (99.8)	JQ857003	*Mrakia robertii* (EF643726)	581/582 (99.8)	*Mrakia robertii*
1	T18	JQ857028	*Mrakia blollopis* (AY038828)	624/624 (100)	JQ857004	*Mrakia blollopis* (AY038828)	555/556 (99.8)	*Mrakia blollopis*
1	T2	JQ857031	*Cryptococcus watticus (*FJ473373*)*	448/448 (100%)	JQ857007	*Holtermanniella watticus* (FJ748666)	368/368 (100)	*Holtermanniella watticus*
1	T29	JQ857033	*Dioszegia crocea* (AF444406)	447/449 (99.6)	JQ857009	*Dioszegia crocea* (HQ256888)	608/612 (99.3)	*Dioszegia sp*
1	T29	JQ857034	*Leucosporidium drummii* (FN908919)	590/620 (95.2)	JQ857010	*Leucosporidiella fragaria* (DQ513270)	583/583 (100)	*Leucosporidiella fragaria*
1	T9	JQ857038	*Dioszegia fristingensis* (EU070927)	462/472 (97.9)	JQ857014	*Dioszegia fristingensis* (JN400789)	445/445 (100)	*Dioszegia fristingensis*
1	T11	JQ857039	*Dioszegia fristingensis* (EU070927)	474/491 (96.5)	JQ857014	*Dioszegia fristingensis* (JN400789)	445/445 (100)	*Dioszegia fristingensis*
2	T2, T18	JQ857025	*Cryptococcus victoriae* (HQ717406)	504/506 (99.6)	JQ857001	*Cryptococcus victoriae* (JN544032)	608/608 (100)	*Cryptococcus victoriae*
2	T8, T32	JQ857032	*Rhodotorula glacialis* (EF151250)	573/573 (100)	JQ857008	*Rhodotorula glacialis* (EF643741)	598/599 (99.8)	*Rhodotorula glacialis*
2	T3, T23	JQ857035	*Mrakia gelida* (AF144485)	622/623 (99.8)<br>622/623 (99.8)	JQ857011	*Mrakia robertii* (EF643731)<br>*Mrakia frigida* (DQ513285)	581/583 (99.7)<br>581/583 (99.7)	*Mrakia sp.*
2	H3, T29	JQ857036	*Mrakia gelida* (GQ911545)	589/590 (99.8)	JQ857012	*Mrakia gelida* (GQ911518)	543/543 (100)	*Mrakia gelida*
2	T11, T20	JQ857037	*Rhodotorula glacialis* (EF151250)	584/588 (99.3)	JQ857013	*Rhodotorula glacialis* (AB671326)	599/602 (99.5)	*Rhodotorula glacialis*
2	T17	JQ857040	*Pseudeurotium bakeri* (GU934582)	512/535 (95.7)	JQ857015	*Leuconeurospora pulcherrima* (FJ176884)	564/578 (97.6)	*Leuconeurospora sp.*
2	T11, T27	JQ857041	*Pseudeurotium bakeri* (GU934582)	514/535 (96.1)	JQ857016	*Leuconeurospora pulcherrima* (FJ176884)	563/579 (97.2)	*Leuconeurospora sp.*
3	H1, H5	JQ857021	*Wickerhamomyces anomalus* (JF416789)	505/505 (100)	JQ856997	*Wickerhamomyces anomalus* (JN180956)	587/587 (100)	*Wickerhamomyces anomalus*
3	T17	JQ857030	*Cryptococcus gilvescens* (AF444380)	613/614 (99.8)	JQ857006	*Cryptococcus gilvescens* (EF643719)	557/557 (100)	*Cryptococcus gilvescens*
4	T5, T19, T14, T26	JQ857018	*Mrakia psychrophila* (EU224267)	595/595 (100)	JQ856994	*Mrakia psychrophila* (EU224266)	596/596 (100)	*Mrakia psychrophila*
4	T8, T10, T13, T27	JQ857029	*Rhodotorula laryngis* (AB078500)	559/562 (99.5)	JQ857005	*Rhodotorula laryngis* (DQ640477)	515/517 (99.6)	*Rhodotorula laryngis*
6	T9, T10, T15, T21, T30	JQ857017	*Glaciozyma antarctica* (AY033637)	579/579 (100)	JQ856993	*Glaciozyma antarctica* (AY040642)	582/583 (99.8)	*Glaciozyma antarctica*
9	T13, T14, T33	JQ857019	*Leucosporidiella creatinivora* (AF444629)	600/601 (99.8)	JQ856995	*Leucosporidiella creatinivora* (AF189925)	611/612 (99.8)	*Leucosporidiella creatinivora*
24	T1, T5, T13, T14, T16, T19, T21, T27, T29, T31, T32, T34	JQ857020	*Sporidiobolus salmonicolor* (AF444611)	544/544 (100)	JQ856996	*Sporidiobolus salmonicolor* (EU596439)	597/597 (100)	*Sporidiobolus salmonicolor*
