# Supplementary figures and images for: Diversity and extracellular enzymatic activities of yeasts isolated from King George Island, the sub-Antarctic region
Source: BMC Microbiol. 2012 Nov 6;12:251. doi: 10.1186/1471-2180-12-251 (PMC3499239; doi:10.1186/1471-2180-12-251)

**T17Cd1**

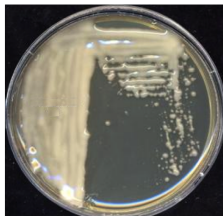

**T11Cd2**

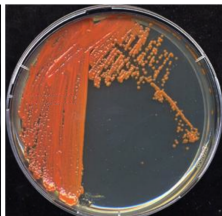

**T27Cd2**

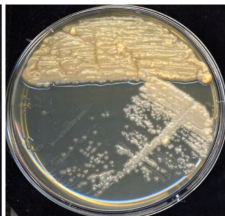

Supplement: Additional file 2 — Colony morphology of Leuconeurospora sp . isolates. Yeasts were cultivated on YM plates supplemented with glucose. The isolates T11Cd2 and T27Cd2 possess identical D1/D2 and ITS sequences, yet are morphologically different. [file 1471-2180-12-251-S2.pdf]
