# Supplementary material for: Diversity and extracellular enzymatic activities of yeasts isolated from King George Island, the sub-Antarctic region
Source: BMC Microbiol. 2012 Nov 6;12:251. doi: 10.1186/1471-2180-12-251 (PMC3499239; doi:10.1186/1471-2180-12-251)
Supplement: Additional file 3 — Carbon source assimilation by yeast isolates obtained in this work. Determinations were performed using the API ID 32C gallery (bioMérieux, Lyon, France) according to manufacturer′s instructions. Gal, D-galactose; Sac, D-sucrose; Nag, N-acetyl-glucosamine; Lat, lactic acid; Ara, L-arabinose; Cel, D-cellobiose; Raf, D-raffinose; Mal, maltose; Tre, D-trehalose; 2kg, 2-ketoglutamate; Mdg, Methyl-αD-glucopiranoside; Man, D-mannitol; Lac, D-lactose; Ino, Inositol; Sor, D-sorbitol; Xyl, D-xylose; Rib, D- ribose; Gly, Gycerol; Rha, L-rhamnnose; Ple, pallatinose; Ery, erytritol; Mel, mellibiose; Grt, glucoronate; Mlz, D-mellicitose; Gnt, gluconate; Lvt, levulinic acid; Glu, D-glucose; Sbe, L-sorbose; Gln, glucosamine. +, assimilation; -, no assimilation. Determinations for each yeast were performed twice. [file 1471-2180-12-251-S3.pdf]

Carbon source assimilation by yeast isolates obtained in this work.

Yeast	Carbon sources																											
	Gal	Sac	Nag	Lat	Ara	Cel	Raf	Mal	Tre	2kg	Mdg	Man	Lac	Ino	Sor	Xyl	Rib	Gly	Rha	Ple	Ery	Mel	Grt	Mlz	Gnt	Lvt	Glu	Sbe	Gln
C. sake	+	+	+	-	-	-	+	+	+	+	+	+	-	-	+	+	-	+	-	+	-	-	-	+	-	+	+	+	+
Cr. gastricus	+	-	+	+	+	+	-	+	+	+	-	-	-	+	+	+	+	+	+	+	-	-	+	+	+	-	+	-	+
Cr. gilvescens	+	+	-	-	+	+	+	+	-	+	-	-	-	+	-	+	-	-	-	-	-	-	+	+	-	-	+	-	-
Cr. victoriae	+	+	+	+	+	+	+	+	+	+	+	+	+	+	+	+	+	+	+	+	+	+	+	+	+	-	+	-	+
Cryptococcus sp.	+	+	+	-	-	+	+	+	-	+	+	-	-	+	+	+	+	-	-	+	-	+	+	+	+	-	+	-	-
D. fristingensis	+	+	+	+	+	+	+	+	+	+	-	+	+	-	+	+	+	+	+	+	-	+	+	+	+	-	+	-	+
Dioszegia sp.	+	+	+	-	+	+	-	+	+	+	-	+	-	-	+	+	+	-	+	+	-	-	+	+	+	-	+	-	-
G. antarctica	-	-	-	-	-	-	-	-	-	-	-	-	-	-	-	-	-	-	-	-	-	-	-	-	-	-	+	-	-
H. watticus	-	-	-	+	+	-	-	+	+	+	-	+	-	-	-	-	-	+	-	-	-	-	-	-	+	-	+	-	+
Le. creatinivora	-	+	+	-	-	-	+	+	+	+	-	+	-	-	+	+	+	+	-	+	-	-	+	+	+	-	+	+	-
Le. fragaria	+	+	+	-	+	+	+	+	+	+	-	+	-	-	-	+	+	+	-	+	-	-	+	+	-	-	+	+	+
Leuconeurospora sp. T11Cd2	+	+	+	-	+	+	+	+	+	+	-	+	-	-	+	+	+	-	+	+	-	+	+	+	+	-	+	-	-
Leuconeurospora sp. T17Cd1	+	+	+	+	+	+	-	+	-	+	-	-	-	-	+	+	+	-	+	+	-	+	+	+	-	-	+	+	+
Leuconeurospora sp. T27Cd2	+	-	+	+	+	+	-	+	+	+	-	-	-	-	+	+	+	-	+	+	-	+	+	+	-	-	+	+	+
M. blollopis	+	+	-	-	+	+	+	+	-	+	-	-	-	-	+	+	+	+	-	-	-	+	+	+	+	-	+	+	-
M. gelida	+	-	+	-	+	+	+	+	+	+	-	+	-	+	+	+	+	-	+	+	-	+	+	+	+	-	+	+	+
M. psychrophila T19Mp	+	+	+	-	+	+	+	+	+	+	-	+	-	+	+	+	-	+	-	-	-	+	+	+	+	-	+	+	+
M. psychrophila T5Mp	+	+	+	-	+	+	+	+	+	+	-	+	-	+	+	+	-	+	+	-	-	+	+	+	+	-	+	+	+
M. robertii	+	+	+	+	+	+	+	+	+	+	-	+	-	+	+	+	+	-	+	+	+	+	+	+	+	-	+	+	+
Mrakia sp.	+	+	+	+	+	+	+	+	+	+	+	+	-	-	+	+	+	+	+	+	+	+	+	+	+	+	+	+	+
Metschnikowia sp.	+	+	+	-	-	+	-	+	+	-	+	+	-	-	+	+	+	+	-	+	-	-	-	+	-	-	+	+	+
Rh. glacialis	+	+	+	-	+	-	+	-	+	+	-	+	-	+	+	+	+	-	+	-	+	+	+	+	+	+	+	+	+
Rh. glacialis	+	+	+	+	+	+	+	+	+	+	+	+	-	-	+	+	+	+	+	+	-	+	+	+	-	+	+	+	+
Rh. laryngis	-	+	+	-	+	-	-	-	+	+	-	+	-	-	+	+	-	+	-	-	-	-	+	+	+	-	+	-	-
Sp. salmonicolor	-	+	-	-	-	-	+	-	+	-	-	+	-	-	+	-	-	-	-	-	-	-	-	-	+	-	+	-	-
W. anomalus	-	+	-	+	-	-	+	+	+	-	+	+	-	-	+	+	-	+	-	+	+	-	-	+	-	-	+	-	-

Determinations were performed using the API ID 32C gallery (bioMérieux, Lyon, France) according to manufacturer´s instructions. Gal, D-galactose; Sac, D-sucrose; Nag, N-acetyl-glucosamine; Lat, lactic acid; Ara, L-arabinose; Cel, D-cellobiose; Raf, D-raffinose; Mal, maltose; Tre, D-trehalose; 2kg, 2-ketoglutamate; Mdg, Methyl-αD-glucopiranoside; Man, D-mannitol; Lac, D-lactose; Ino, Inositol; Sor, D-sorbitol; Xyl, D-xylose; Rib, D- ribose; Gly, Gycerol; Rha, L-rhamnnose; Ple, pallatinose; Ery, erytritol; Mel, mellibiose; Grt, glucoronate; Mlz, D-mellicitose; Gnt, gluconate; Lvt, levulinic acid; Glu, D-glucose; Sbe, L-sorbose; Gln, glucosamine. +, assimilation; -, no assimilation. Determination for each yeast were performed twice.
